# Publisher Correction: Dynamic mRNA degradome analyses indicate a role of histone H3K4 trimethylation in association with meiosis-coupled mRNA decay in oocyte aging

**DOI:** 10.1038/s41467-022-31224-4

**Published:** 2022-06-15

**Authors:** Yun-Wen Wu, Sen Li, Wei Zheng, Yan-Chu Li, Lu Chen, Yong Zhou, Zuo-Qi Deng, Ge Lin, Heng-Yu Fan, Qian-Qian Sha

**Affiliations:** 1 MOE Key Laboratory for Biosystems Homeostasis & Protection and Innovation Center for Cell Signaling Network, Life Sciences Institute, Zhejiang University, 310058 Hangzhou, China; 2 Fertility Preservation Laboratory, Reproductive Medicine Center, Guangdong Second Provincial General Hospital, 510317 Guangzhou, China; 3 Research Center for Reproduction and Genetics in Hunan Province, Reproductive and Genetic Hospital of CITIC-XIANGYA, 410008 Changsha, China; 4 The Second School of Clinical Medicine, Southern Medical University, 510515 Guangzhou, China; 5 School of Basic Medical Sciences, Southern Medical University, 510515 Guangzhou, China

**Keywords:** Oogenesis, Infertility, RNA decay, Epigenetics

Correction to: *Nature Communications* 10.1038/s41467-022-30928-x, published online 09 June 2022.

In this article the affiliation details for Yan-Chu Li, Yong Zhou and Qian-Qian Sha were incorrectly given as “MOE Key Laboratory for Biosystems Homeostasis & Protection and Innovation Center for Cell Signaling Network, Life Sciences Institute, Zhejiang University, 310058 Hangzhou, China” but should have been “Fertility Preservation Laboratory, Reproductive Medicine Center, Guangdong Second Provincial General Hospital, 510317 Guangzhou, China”. The affiliation details for Lu Chen, Zuo-Qi Deng and Heng-Yu Fan were incorrectly given as “Fertility Preservation Laboratory, Reproductive Medicine Center, Guangdong Second Provincial General Hospital, 510317 Guangzhou, China” but should have been “MOE Key Laboratory for Biosystems Homeostasis & Protection and Innovation Center for Cell Signaling Network, Life Sciences Institute, Zhejiang University, 310058 Hangzhou, China”. The original article has been corrected.

