# Supplementary material for: Dynamic mRNA degradome analyses indicate a role of histone H3K4 trimethylation in association with meiosis-coupled mRNA decay in oocyte aging
Source: Nat Commun. 2022 Jun 9;13:3191. doi: 10.1038/s41467-022-30928-x (PMC9184541; doi:10.1038/s41467-022-30928-x)
Supplement: Supplementary file 1 — Supplementary Information [file 41467_2022_30928_MOESM1_ESM.pdf]

Supplementary Figures

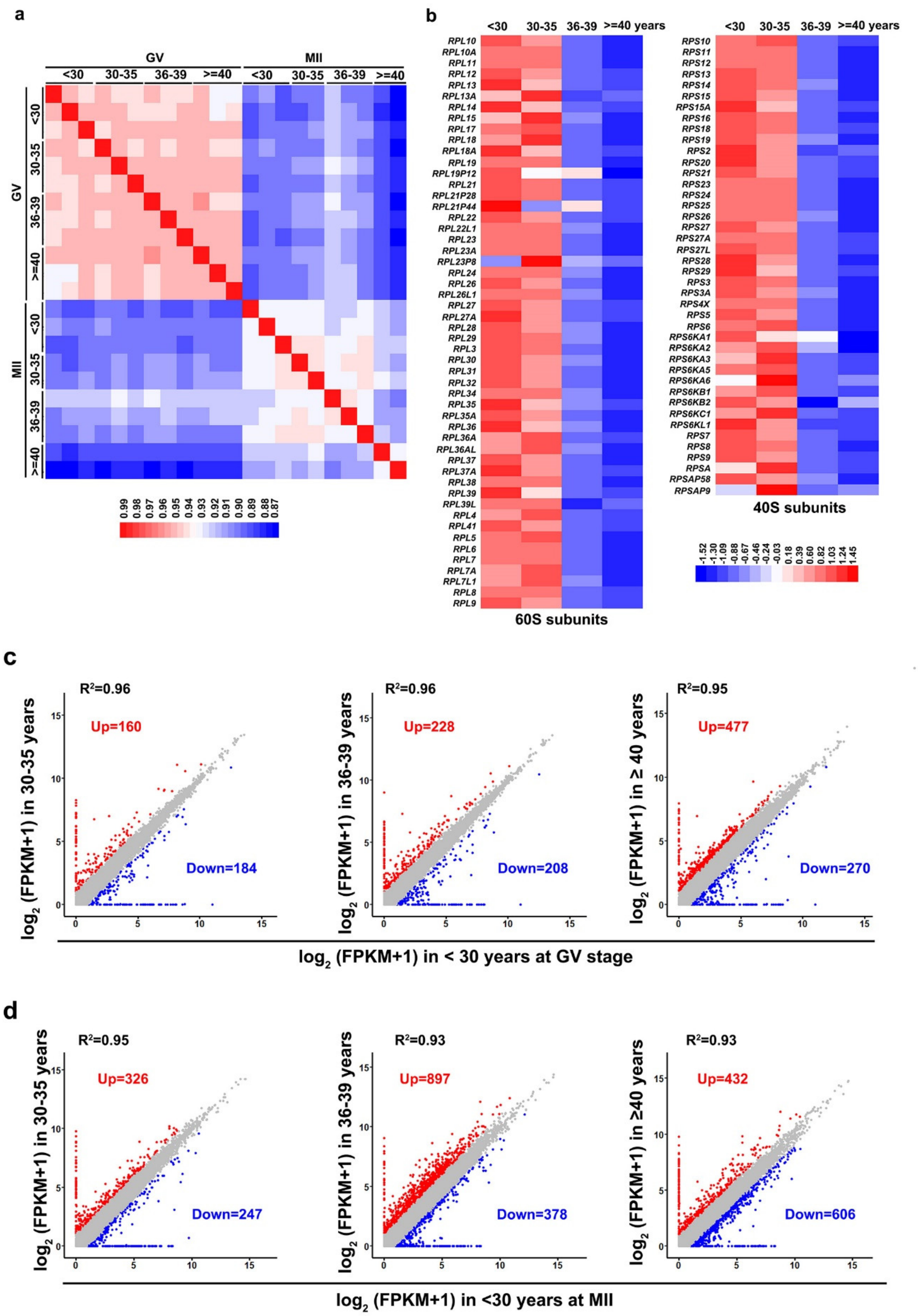

Figure S1: Age-associated transcriptome changes in human oocytes.

a: Heatmap of Spearman correlation coefficients of total transcripts among the indicated samples in humans. **b:** Heat maps showing the level changes in transcripts encoding ribosome subunits in GV oocytes of women with different ages. **c:** Scatter plots of RNA-seq data illustrating transcriptional changes in human GV oocytes of different ages without ERCC spike-in normalization. Transcripts decreased or increased by more than 2-fold compared with oocytes of women younger than 30 years old were highlighted with different colors. **d:** Scatter plots of RNA-seq data illustrating the transcriptional changes in human MII oocytes of different ages without ERCC spike-in normalization. Transcripts that decreased or increased by more than 2-fold compared with oocytes of women younger than 30 years old are highlighted with different colors.

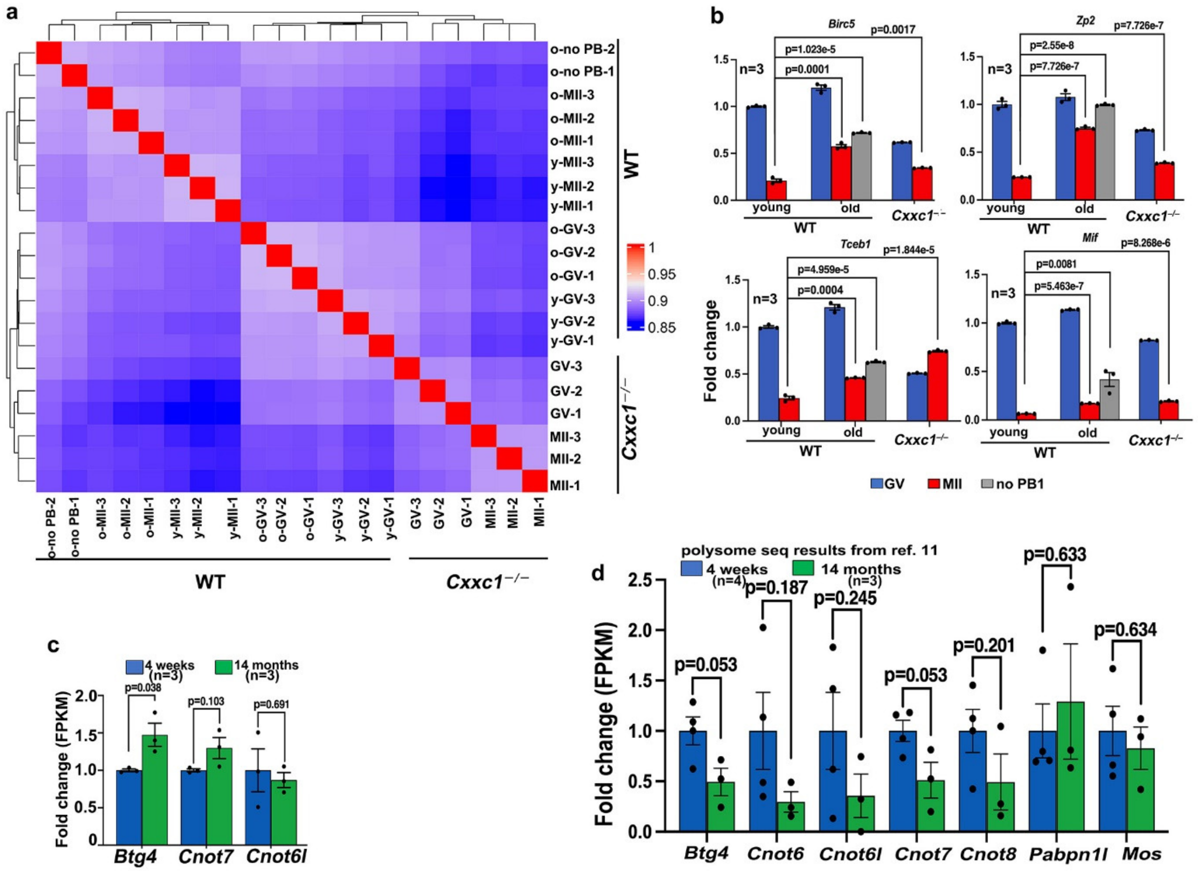

Figure S2: Aging-associated transcriptome changes in WT and *Cxxc1*-deleted oocytes.

a: Principal component analysis (PCA) results of indicated samples. **b:** RT-qPCR results showing the mRNA levels of selected M-decay transcripts in WT (4-week and 14-month) and *Cxxc1*-deleted oocytes (4-week) before and after *in vitro* maturation culture. Data are presented as mean values ± SEM. *P* by two-tailed Student's t-test. n = 3 biological replicates. **c:** FPKM values of indicated transcripts extracted from RNA-seq results described in Fig. 1B. Data are presented as mean values ± SEM. *P* by two-tailed Student's *t*-test. n = 3 biological replicates. **d:** Polysome isolation and sequencing results extracted from original data of reference 11 showing the potential translational activity of maternal transcripts encoding subunits of CCR4-NOT in young and aged mouse oocytes. Polysome isolation and sequencing results of *Mos* transcript was also presented as a comparison, which does not show a significant change in the aged oocytes. Data are presented as mean values ± SEM. *P* by two-tailed Student's *t*-test. n = 4 biological replicates in oocytes of 4 weeks old mice; n = 3 biological replicates in oocytes of 14 months old mice.

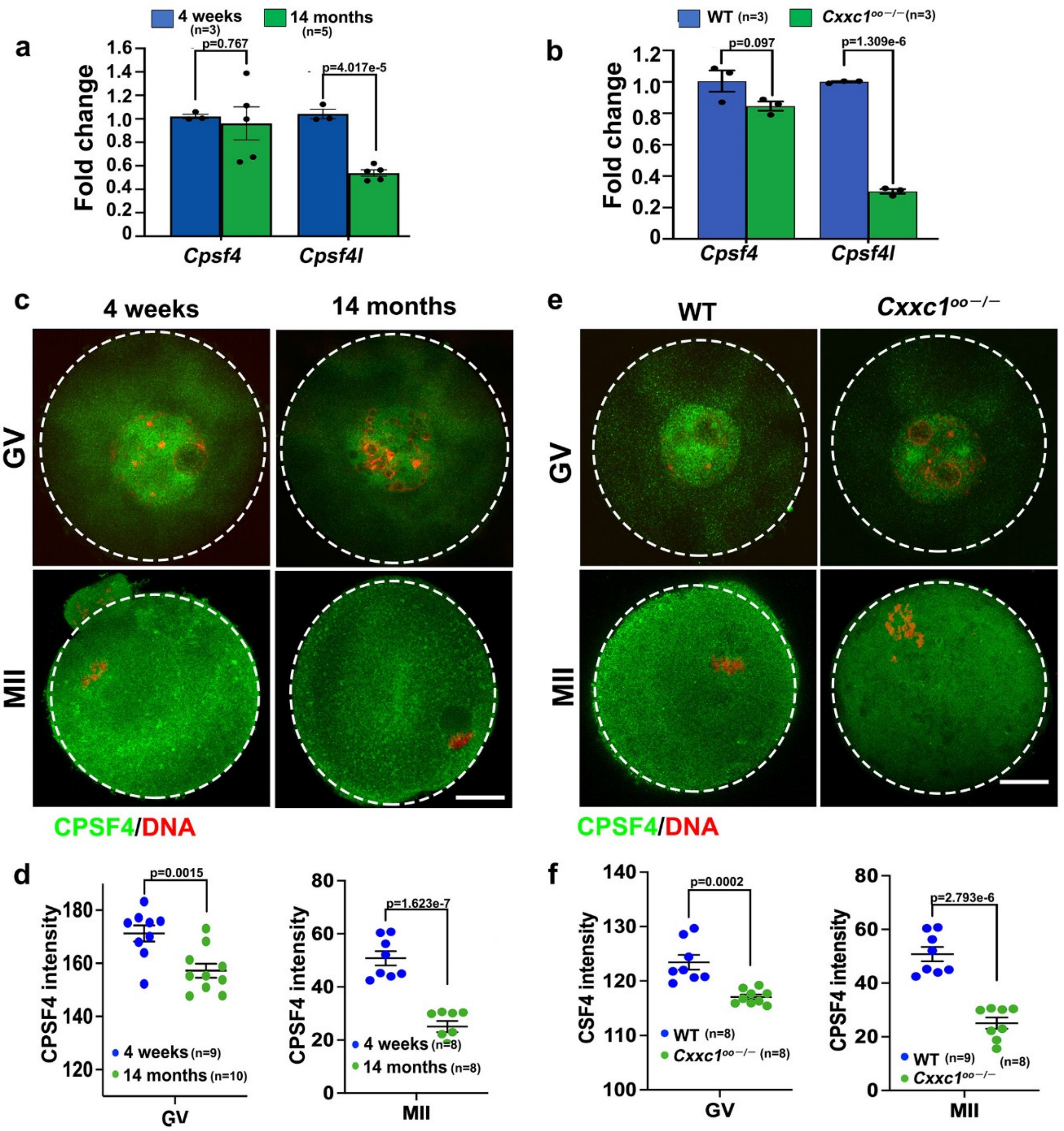

Figure S3. Expression of CPSF4 and CPSF4L in mouse oocytes. a: Quantitative RT-PCR results showing the expression of *Cpsf4* and *Cpsf4l* mRNAs in GV oocytes of 4-week and 14-month old WT mice. n = 3 biological replicates in oocytes of 4 weeks old mice; n = 5 biological replicates in oocytes of 14 months old mice. **b:** Quantitative RT-PCR results showing the expression of *Cpsf4* and *Cpsf4l* mRNAs in GV oocytes of 4-week-old WT and *Cxxc1$^{fl/fl}$;Gdf9-Cre* mice. n = 3 biological replicates **c:** Immunofluorescence results showing the expression of CPSF4 protein in GV oocytes of 4-week and 14-month-old WT mice. Scale bar = 10 μm. **d:** Quantification of the immunofluorescence signals in (**c**). **e:** Immunofluorescence results showing the expression of CPSF4 protein in GV oocytes of 4-week-old WT and *Cxxc1$^{fl/fl}$;Gdf9-*

Cre mice. Scale bar = 10 µm. **f:** Quantification of the immunofluorescence signals in (**e**).

In **a, b, d,** and **f**, data are presented as mean values ± SEM. *P* by two-tailed Student's *t*-test. n indicates the number of oocytes for each experimental group.

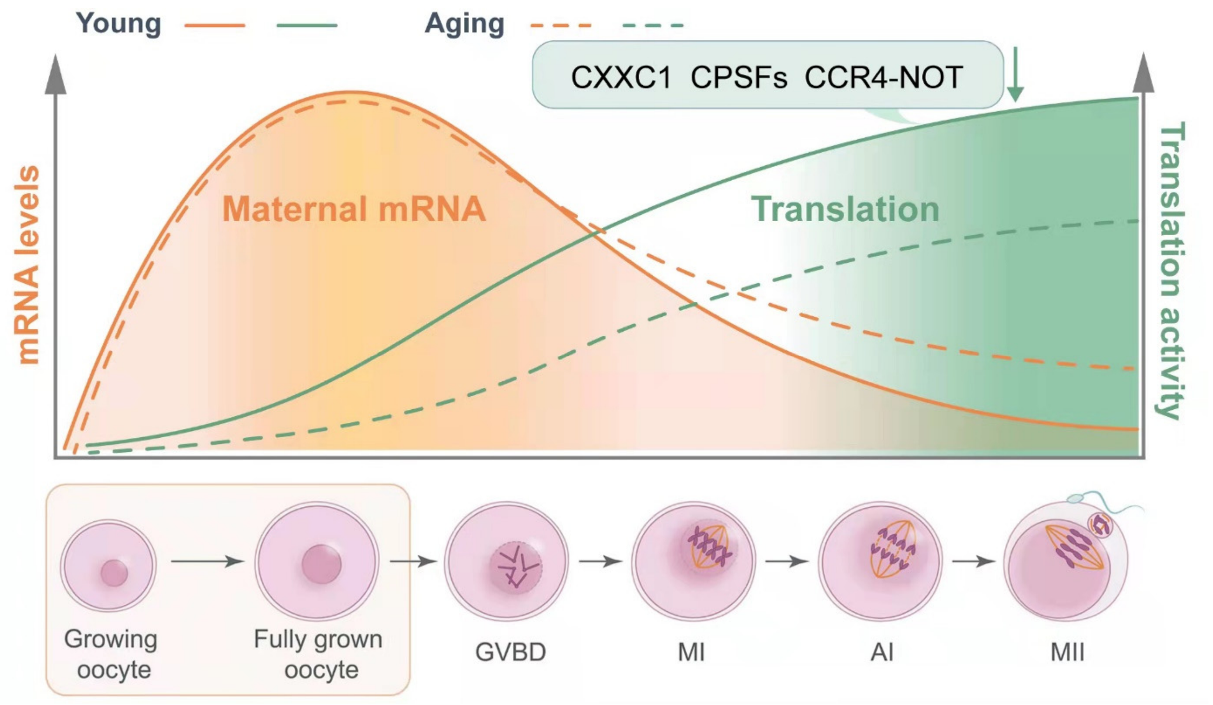

Figure S4. CXXC1-maintained histone H3K4 trimethylation and meiosis-coupled mRNA decay mediate age-associated transcriptome changes in mouse oocytes. Although in aged human GV oocytes, the mRNA showed decreased expression levels compared to young oocytes (Fig 1a and Fig.2e), the mRNA level are comparable in young and aged mouse GV oocytes. However, meiotic maturation-coupled mRNA translation and decay are impaired in the oocytes of aged females. The decline of CXXC1-maintained H3K4me3 level led to inefficiency of mRNA translation, and clearance, anovulation, and ultimately, female infertility.
