# Supplementary material for: Dynamic mRNA degradome analyses indicate a role of histone H3K4 trimethylation in association with meiosis-coupled mRNA decay in oocyte aging
Source: Nat Commun. 2022 Jun 9;13:3191. doi: 10.1038/s41467-022-30928-x (PMC9184541; doi:10.1038/s41467-022-30928-x)
Supplement: Supplementary file 3 — Description of Additional Supplementary Files [file 41467_2022_30928_MOESM3_ESM.docx]

File name: Supplementary Data 1-6

Supplementary Data 1. Spearman correlation coefficients among different human oocytes.

Supplementary Data 2. Spearman correlation coefficients among different mouse oocytes.

Supplementary Data 3. Antibody information

Supplementary Data 4. Primer Sequences

Supplementary Data 5. Quality control of human RNA-seq results.

Supplementary Data 6. Quality control of mouse RNA-seq results.
