# Supplementary material for: Dynamic mRNA degradome analyses indicate a role of histone H3K4 trimethylation in association with meiosis-coupled mRNA decay in oocyte aging
Source: Nat Commun. 2022 Jun 9;13:3191. doi: 10.1038/s41467-022-30928-x (PMC9184541; doi:10.1038/s41467-022-30928-x)
Supplement: Supplementary file 5 — Reporting Summary [file 41467_2022_30928_MOESM5_ESM.pdf]

nature research

Corresponding author(s): Heng-Yu Fan

Last updated by author(s): May 9, 2022

Reporting Summary

Nature Research wishes to improve the reproducibility of the work that we publish. This form provides structure for consistency and transparency in reporting. For further information on Nature Research policies, see Authors & Referees and the Editorial Policy Checklist.

Statistics

For all statistical analyses, confirm that the following items are present in the figure legend, table legend, main text, or Methods section.

n/a	Confirmed	
☐	☒	The exact sample size (*n*) for each experimental group/condition, given as a discrete number and unit of measurement
☐	☒	A statement on whether measurements were taken from distinct samples or whether the same sample was measured repeatedly
☐	☒	The statistical test(s) used AND whether they are one- or two-sided<br>*Only common tests should be described solely by name; describe more complex techniques in the Methods section.*
☒	☐	A description of all covariates tested
☒	☐	A description of any assumptions or corrections, such as tests of normality and adjustment for multiple comparisons
☐	☒	A full description of the statistical parameters including central tendency (e.g. means) or other basic estimates (e.g. regression coefficient) AND variation (e.g. standard deviation) or associated estimates of uncertainty (e.g. confidence intervals)
☐	☒	For null hypothesis testing, the test statistic (e.g. *F*, *t*, *r*) with confidence intervals, effect sizes, degrees of freedom and *P* value noted<br>*Give P values as exact values whenever suitable.*
☒	☐	For Bayesian analysis, information on the choice of priors and Markov chain Monte Carlo settings
☒	☐	For hierarchical and complex designs, identification of the appropriate level for tests and full reporting of outcomes
☒	☐	Estimates of effect sizes (e.g. Cohen's *d*, Pearson's *r*), indicating how they were calculated

Our web collection on statistics for biologists contains articles on many of the points above.

Software and code

Policy information about availability of computer code

Data collection	Image data was collected with LSM 700 and the software, ZEN 2.
Data analysis	Quantification of Western blot was performed by ImageJ2x. Statistics were calculated by Microsoft Excel 2010.RNA-seq data are analysed by Cufflinks (v2.2.1).

For manuscripts utilizing custom algorithms or software that are central to the research but not yet described in published literature, software must be made available to editors/reviewers. We strongly encourage code deposition in a community repository (e.g. GitHub). See the Nature Research guidelines for submitting code & software for further information.

Data

Policy information about availability of data

All manuscripts must include a data availability statement. This statement should provide the following information, where applicable:

- Accession codes, unique identifiers, or web links for publicly available datasets
- A list of figures that have associated raw data
- A description of any restrictions on data availability

Authors can confirm that all relevant data are included in the paper and/or its supplementary information files. RNA-seq data have been deposited in the NCBI Gene Expression Omnibus database under the accession code GSE173598 with the token wretkiuoxteffcf.

Field-specific reporting

Please select the one below that is the best fit for your research. If you are not sure, read the appropriate sections before making your selection.

☒ Life sciences ☐ Behavioural & social sciences ☐ Ecological, evolutionary & environmental sciences

For a reference copy of the document with all sections, see nature.com/documents/nr-reporting-summary-flat.pdf

Life sciences study design

All studies must disclose on these points even when the disclosure is negative.

Sample size	No specific statistical methods were used to predetermine the sample size. According to the experience in our previously published studies (Sha QQ et al, Nature Communications 2020; Sha QQ et al, Nucleic Acids Res 2021) to choose an adequate pool for reporter assay, microscopy, qTR-PCR, and mouse experiments. For each experiment, the samples used were indicated in the figures or legends.
Data exclusions	No data were excluded.
Replication	All results were reproduced at least twice.
Randomization	Only littermates were used for WT control and knockout mice. Samples/organisms/participants of the same genotype/age were randomly allocated into experimental groups.
Blinding	The investigators were blinded to group allocation during oocyte manipulation and RNA-seq data collection and analysis.

Reporting for specific materials, systems and methods

We require information from authors about some types of materials, experimental systems and methods used in many studies. Here, indicate whether each material, system or method listed is relevant to your study. If you are not sure if a list item applies to your research, read the appropriate section before selecting a response.

Materials & experimental systems

n/a	Involved in the study	
☐	☒	Antibodies
☒	☐	Eukaryotic cell lines
☒	☐	Palaeontology
☐	☒	Animals and other organisms
☐	☒	Human research participants
☒	☐	Clinical data

Methods

n/a	Involved in the study	
☒	☐	ChIP-seq
☒	☐	Flow cytometry
☒	☐	MRI-based neuroimaging

Antibodies

Antibodies used	The detailed information of primary and secondary antibodies is described in Supplementary Data 1.
Validation	All primary antibodies are validated for their specificities. The secondary antibodies with minimal cross-reactivities were purchased from Jax ImmunoResearch.

Animals and other organisms

Policy information about studies involving animals; ARRIVE guidelines recommended for reporting animal research

Laboratory animals	The 4-weeks and 14-months old C57BL6 strain female mice were used in this study.
Wild animals	No wild animals used in this study
Field-collected samples	This study did not contain samples derived from animals from the field.
Ethics oversight	All experimental protocols involving mice were approved by the Zhejiang University Institutional Animal Care and Research Committee (Approval # ZJU20170014).

Note that full information on the approval of the study protocol must also be provided in the manuscript.

Human research participants

Policy information about studies involving human research participants

Population characteristics	All human oocytes were collected from woman (< 35 years old; 35-40 years old; 40-45 years old; and > 45 years old) volunteers who seek assisted reproduction at clinic.
Recruitment	Participants were randomly selected. The oocytes from these participants were divided into 4 groups: < 35 years old; 35-40 years old; 40-45 years old; and > 45 years old. There is no potential self-selection bias or other biases that may impact results.
Ethics oversight	The study design and conduct complied with all relevant regulations regarding the use of human study participants and was conducted in accordance with the criteria set by the Declaration of Helsinki. This study have complied with all relevant ethical regulations for work with human participants, and that written informed consent was obtained. The experiments performed in this study were approved and guided by the ethical committee of the Reproductive Medicine Center of Guangdong Second Provincial General Hospital (Research license 20190906-01-03-YXKYYJ-GZRKT) and the Reproductive & Genetic Hospital of CITIC-XIANGYA (Research license LL-SC-2017-012-1).

Note that full information on the approval of the study protocol must also be provided in the manuscript.
