# Supplementary figures and images for: Dynamic mRNA degradome analyses indicate a role of histone H3K4 trimethylation in association with meiosis-coupled mRNA decay in oocyte aging
Source: Nat Commun. 2022 Jun 9;13:3191. doi: 10.1038/s41467-022-30928-x (PMC9184541; doi:10.1038/s41467-022-30928-x)

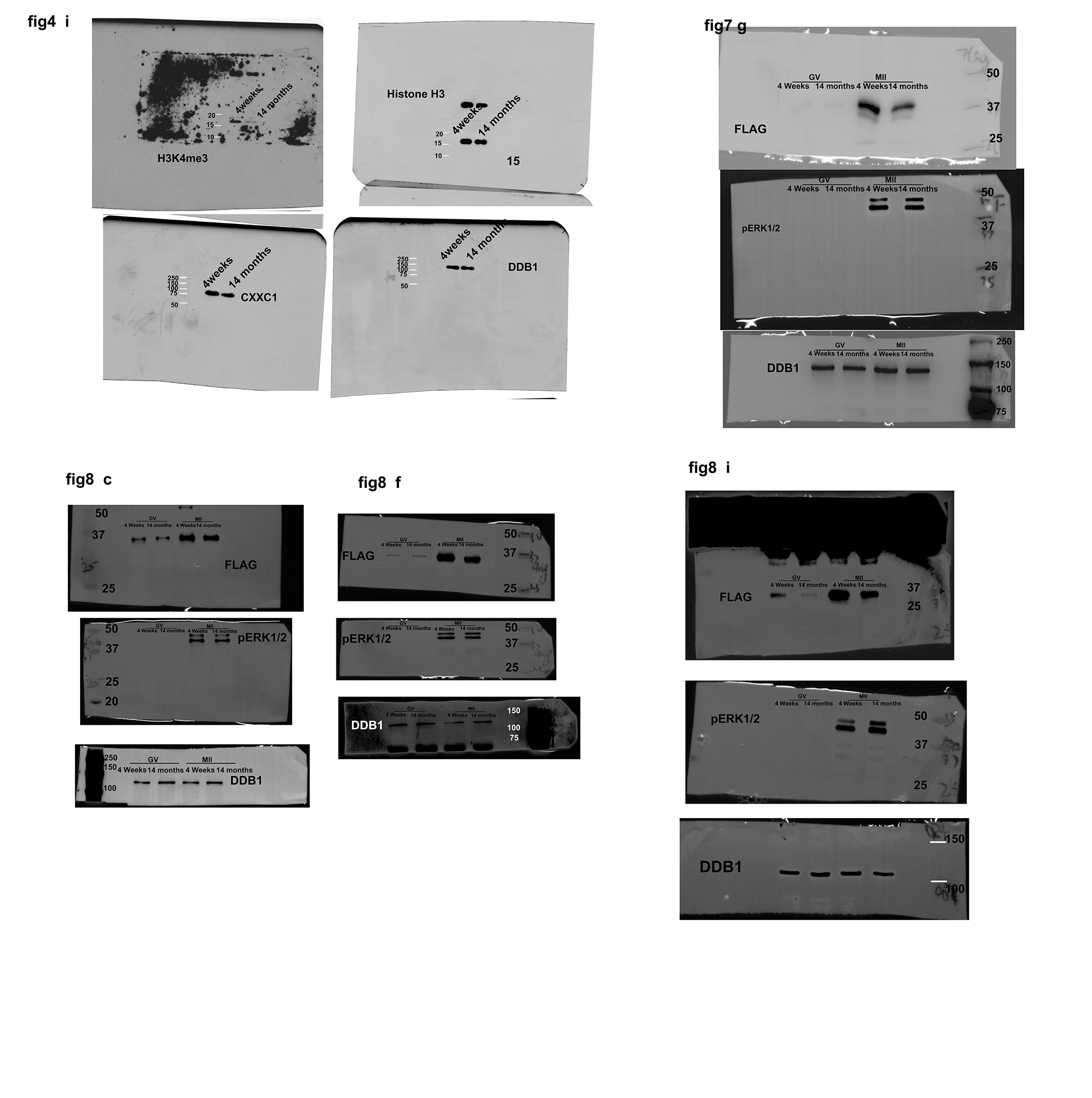

Supplement: Supplementary file 6 — Source Data [file 41467_2022_30928_MOESM6_ESM.jpg]
